# Fe-TAML: Catalyst for Cleanup

**DOI:** 10.1289/ehp.114-a656

**Published:** 2006-11

**Authors:** Valerie J. Brown

Hormones occur naturally in humans, animals, and plants, but estrogens in contraceptives and hormone replacement therapy, those given to agricultural animals, and industrial chemicals that mimic estrogen add to the enormous volume of biologically active compounds in the environment. These compounds are among the PPCP (pharmaceutical and personal care product) category of contaminants that, when excreted, make their way into surface and ground-water, wastewater treatment plants, and, eventually, back into drinking water supplies. Now scientists are looking to catalysts called Fe-TAML^®^ (iron plus tetra-amido macrocyclic ligand) activators as a promising way to remove these contaminants from wastewater.

## The Estrogen Question

Estrogens are among the most potent chemicals in nature; although environmental concentrations of these compounds are typically low, the low dose response of animals to estrogens makes their introduction into the environment a concern. The most potent naturally occurring female hormone is 17β-estradiol; the synthetic version, 17β-ethinylestradiol, is used in birth control pills. According to a paper published in the 1 July 2004 issue of *Environmental Science & Technology*, cows and swine in the United States excrete a daily mix of 10–30 kg of 17β-estradiol and 20–80 kg of estrone, a major metabolite of estrogen—likely over an order of magnitude higher than what the human population puts out.

Disruptions in estrogens’ balance have been linked to a variety of cancers, malformation of reproductive organs, sperm abnormalities, and other consequences in animals. As summarized in a paper in the April 2006 *EHP Ecology Special Issue* by Melanie Gross-Sorokin and others, many studies have reported reproductive abnormalities in fish living in waters downstream of estrogen-containing wastewater discharges in the United States and Europe.

But although there is little disagreement among researchers that estrogenic compounds in the environment can have negative effects in animals, especially on pre- and postnatal developmental processes, there is far less agreement on the type and magnitude of effects in both humans and other organisms from chronic low-level exposures to reproductive hormones and hormone-mimicking chemicals. The type and magnitude of effects from combinations of such compounds also have not yet been clearly elucidated. Thomas White, a technical consultant for the Pharmaceutical Research and Manufacturers of America, says, “Based on preliminary human health assessments for about twenty-six pharmaceutical compounds, we believe there is no appreciable risk to human health from pharmaceuticals in the environment. More research is necessary in order to make this same assessment for potential ecological impacts.”

To measure the magnitude of PPCPs and other emerging contaminants in national waters, the USGS analyzed 139 streams around the United States in 1999 and 2000 for 95 organic wastewater contaminants. Of 13 compounds that affect reproduction, including estrogenic and androgenic compounds, 1 or more were found at 37% of the sites. 17β-ethinylestradiol was found in 5.7% of samples, 17β-estradiol in 10%. The median detectable concentration of 17β-ethinylestradiol was 0.094 μg/L, of 17β-estradiol, 0.009 μg/L. The sites included mostly areas with probable contamination from human, industrial, and agricultural wastewater, so these findings should not be considered representative of all streams in the United States, according to the report, which appeared in the 15 March 2002 issue of *Environmental Science & Technology*.

The report observes that even though nonprescription drugs were found in much greater concentrations, “measured concentrations of reproductive hormones may have greater implications for health of aquatic organisms” because these compounds can have deleterious effects at exposures in the range of 0.001 μg/L. The USGS findings suggested that some environmental exposures are above levels where effects have been observed in fish. The USGS report also states that the presence of organic wastewater contaminants at the national scale observed in the study implies that current wastewater treatment and natural biodegradation are inadequate to completely remove them from water.

Thus, an effective method of removing estrogens, their metabolites, and chemical mimics may become more important as the science evolves, and wastewater treatment plants and drinking water systems may eventually be required to treat source waters for estrogens. The EPA announced in August 2005 that some PPCPs may be added to its next contaminant candidate list, a means of prioritizing research goals with the possibility of eventual regulation. The next list is expected in 2008.

## The Fe-TAML Approach

Fe-TAMLs were first developed by a research team led by Terrence Collins at Carnegie Mellon University’s Institute for Green Oxidation Chemistry. The elegant Fe-TAML molecule features an iron atom in its center, surrounded by four nitrogen atoms, which in turn are corralled by a ring of carbon atoms. Water molecules can loosely attach to the vertical pole of the iron atom as ligands. If hydrogen peroxide is present, it can displace a water ligand and create a catalyst that triggers oxidation reactions with other compounds in the solution.

In studies presented on 29 June 2006 at the Green Chemistry and Engineering Conference in Washington, DC, Nancy Shappell, a research physiologist with the USDA Agricultural Research Service in Fargo, North Dakota, demonstrated that these catalysts can work with hydrogen peroxide to rapidly break down 17β-estradiol and 17β-ethinylestradiol. Depending on temperature and other conditions, 17β-estradiol has a natural half-life of about a week, whereas 17β-ethinylestradiol takes about twice that time. In Shappell’s laboratory experiments, developed in collaboration with the Institute for Green Oxidation Chemistry, the Fe-TAML process neutralized trace quantities of both estrogens in a mere five minutes. In work first reported in the January–February 2006 issue of the *Journal of Environmental Quality*, Shappell has also begun quantifying the background levels of estrogenic activity of surface waters in order to put the anthropogenic contributions in context.

The orange Fe-TAML catalyst is first dissolved in water and then added to the solution containing the target chemical. It works at room temperature and up to at least 90°C, according to team member Colin Horwitz, a Carnegie Mellon research professor. The group has found that Fe-TAMLs together with hydrogen peroxide can rapidly degrade not only estrogenic compounds, but also bacterial spores similar to those of anthrax, sulfur compounds in motor fuels, dyes in textile mill wastewater, and organic colorants discharged from pulp and paper mills. Fe-TAML catalyst processes also degrade organophosphorus pesticides as well as their toxic degradation intermediates, some of which have been implicated as endocrine disruptors, says Horwitz. However, at the current stage of development, Fe-TAML activators work poorly with polychlorinated biphenyls.

Shappell cautions that although the Fe-TAML catalyst process “is very effective at destroying synthetic estradiol and also several other estrogens, whether it’s going to be effective in an organic matrix such as wastewater is another issue that we haven’t evaluated yet.” Currently the Fe-TAML technology is somewhere between the proof-of-principle stage of investigation and the small-scale pilot plant stage of development, according to Horwitz.

He expects the base catalyst to be available in “small ton quantities” within about two years, adding that the technology may initially be a niche technology before it becomes applicable in large wastewater facilities. However, the catalyst appears to be very efficient. In the pulp and paper application—the only one thus far to use actual wastewater instead of an isolated target chemical in the laboratory—2 metric tons of the catalyst could treat a year’s worth of pulp from a mill producing 300 metric tons per day, says Horwitz.

## A Pipe Dream?

People on the front lines of water treatment tend to be skeptical of contaminant cleanup claims that sound too good to be true, although among the treatment community there is great interest in the Fe-TAML technology. Portland, Oregon’s municipal water treatment department participated in the USGS survey, and Chuck Lytle, manager of Portland’s Water Pollution Control Laboratory, calls emerging contaminants including hormones an “extremely new and hot topic.” But the city is not yet gearing up to treat for PPCPs. “We are not proactive,” Lytle observes, because waterworks companies and city bureaus generally wait for the EPA and state environmental quality departments to lay down the fiats regarding removal of additional contaminants.

The Carnegie Mellon team aims to create thoroughly green chemistry—that is, chemicals and processes that don’t create more problems than they solve. Theo Colborn, president of the Colorado-based Endocrine Disruption Exchange, says, “I think it’s exciting what they’re trying to do” but asks, “do they really know what they’re breaking [compounds] into?” In some cases bacteria will reconstruct the metabolites back into the original compound. Similarly, Christian Daughton, chief of the Environmental Chemistry Branch at the EPA National Exposure Research Laboratory, says there are “other solutes that are also present at the same time, most of which may not even be characterized and which will also engage in oxidative reactions” in the presence of the catalyst.

However, according to Horwitz, tests have shown that the catalyst itself is non-toxic to aquatic biota, and the degradation by-products are also benign. In the 12 April 2002 issue of *Science* and the January 2006 issue of the *Journal of the American Chemical Society*, the Carnegie Mellon team published the breakdown profile for pollutants such as pentachlorophenol, 2,4,6-trichlorophenol, and fenitrothion, where Fe-TAML processes appeared to provide an effective, nontoxic approach.

Horwitz agrees that concerns about creating unintended products are valid, especially since “all waste streams are different, and they can change over a very short time.” Carnegie Mellon has conducted collaborative studies with New Zealand researchers L. James Wright, a professor at the University of Auckland, and Trevor Stuthridge at biomedical research institute Scion Research with the Fe-TAML/H2O2 system. “We have now done toxicity testing after treatment of some pesticides, chlorinated phenols, bisphenol A, some organic dyes, and some pharmaceuticals, and in all cases we find that the toxicity of the treated sample is lower than the untreated samples,” Horwitz says. “In some cases, it appears as though toxicity after treatment is essentially zero.”

Many wastewater treatment plants rely on biological degradation of contaminants by bacteria, but this is often not effective for all contaminants. Membrane filtration, including reverse osmosis, is a proven way to remove the majority of chemicals, but it is energy-intensive and expensive, says Jean Debroux, a scientist/engineer with the San Francisco–based engineering and environmental sciences consulting firm Kennedy/Jenks. In addition, Debroux says, “For every two gallons of water you treat with reverse osmosis membranes, you end up with approximately one gallon of [treated] water and one gallon of the rejects, which concentrates everything in that water. So now you have to do something with that.” Thus, if the speed of estrogen degradation by the Fe-TAML catalyst holds up in large-scale applications without generating further toxic compounds, it would be a major boon to wastewater treatment.

In terms of potential effects, Shappell feels the next step should be to characterize the fate of estrone, which she says is present in wastewater in “much higher concentrations” than 17β-estradiol. The Fe-TAML catalyst degraded estrone about as efficiently as the estradiol in Shappell’s test of the catalyst. Estrone’s potency ranges from one-tenth to one-hundredth that of 17β-estradiol, depending on species and assay. “While estradiol has been the focus of environmental measurements,” Shappell says, “typically the waste has very little once it has sat around any length of time, and in fact estrone is the primary player. I think someone needs to do generational studies on fish to determine concentration of estrone that results in sterile males.”

The Fe-TAML catalyst seems to offer almost magical solutions to a variety of wastewater problems, but much refinement of the process remains to be done, and not all stakeholders are yet convinced such treatment is necessary, especially with respect to PPCPs. But in a world where the environmental news is all too often disheartening, Fe-TAMLs offer a glow of hope that the massive power of chemistry can mitigate some of the problems of modern life.

## Figures and Tables

**Figure:**
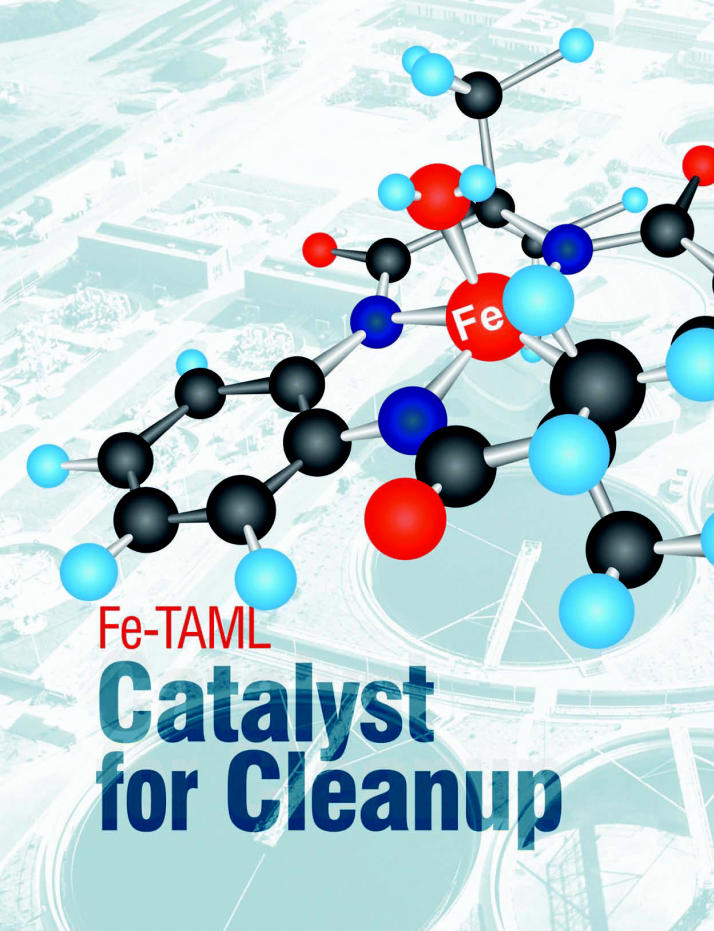


**Figure:**
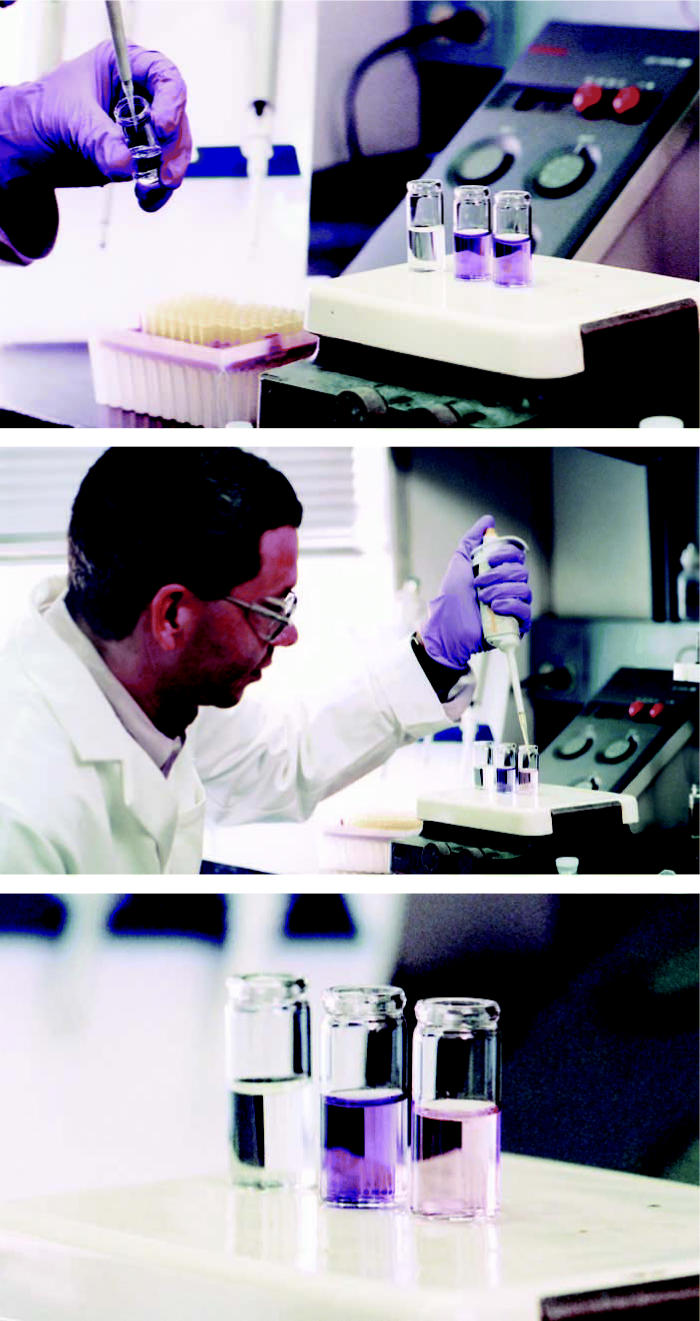
Now you see it, now you don’t (top) Textile dye has been added to two of three vials of water. (middle) Colin Horwitz adds the Fe-TAML activator and hydrogen peroxide to the third vial, which contains dye. (bottom) The catalyst removes the dye, leaving the liquid clear.

**Figure:**
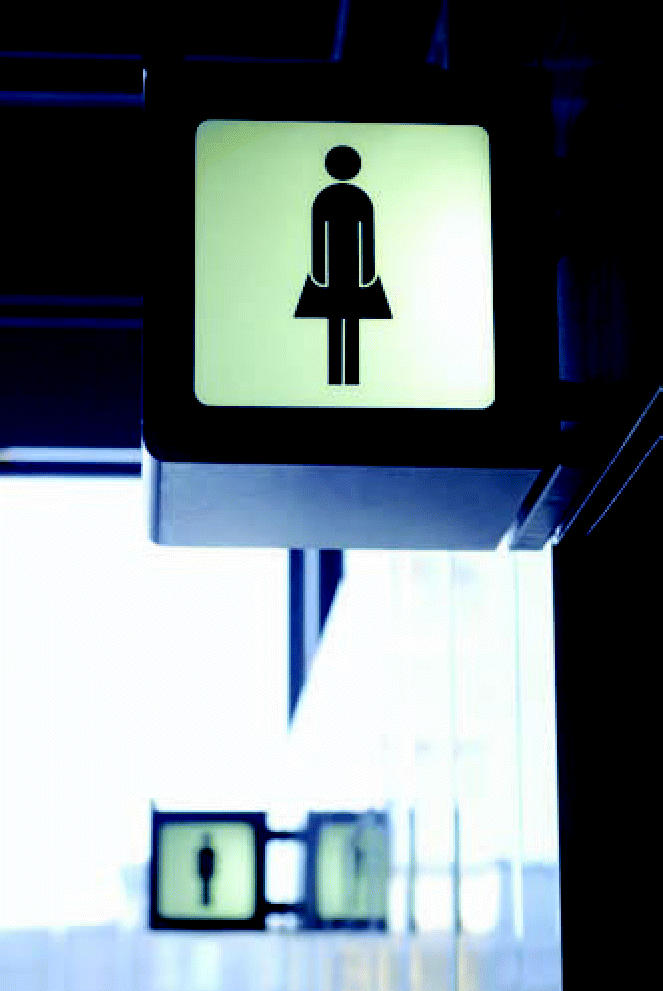
Point source pollution? Synthetic estrogens are excreted into the waste stream and make their way into the environment.
